# Categorization of Opioid Use Among Pregnant People and Association With Overdose or Death

**DOI:** 10.1001/jamanetworkopen.2022.14688

**Published:** 2022-05-27

**Authors:** Andi Camden, Teresa To, Joel G. Ray, Tara Gomes, Li Bai, Astrid Guttmann

**Affiliations:** 1 Dalla Lana School of Public Health, University of Toronto, Toronto, Ontario, Canada; 2 ICES, Toronto, Ontario, Canada; 3 The Hospital for Sick Children, Toronto, Ontario, Canada; 4 Department of Obstetrics and Gynaecology, St Michaels Hospital, Toronto, Ontario, Canada; 5 Li Ka Shing Knowledge Institute, St Michael’s Hospital, Toronto, Ontario, Canada; 6 Institute of Health Policy, Management and Evaluation, University of Toronto, Toronto, Ontario, Canada; 7 Edwin SH Leong Centre, University of Toronto, Toronto, Ontario, Canada

## Abstract

**Importance:**

Early identification of people who use opioids in pregnancy may improve health outcomes for pregnant people and infants. However, characterization of diverse circumstances surrounding type of opioid use and indications for opioid use are lacking.

**Objective:**

To develop clinically distinct groups of people who use opioids in pregnancy and to evaluate their association with drug overdose or death up to 1 year post partum.

**Design, Setting, and Participants:**

This is a population-based, repeated cross-sectional study conducted in Ontario, Canada, with participants who used opioids in pregnancy who had a live birth or stillbirth between January 1, 2014, and December 31, 2019, identified in health administrative databases. Data were analyzed from August 2020 to January 2021.

**Exposures:**

Prenatal opioid use.

**Main Outcomes and Measures:**

Latent class analysis (LCA), based on prenatal opioid use and 19 socioeconomic and medical characteristics, first identified clinically distinct groups of opioid users. Then, within the optimally derived LCA-derived group, adjusted relative risks (aRRs) were generated for the outcome of drug overdose or all-cause death within 1 year post partum, adjusting for birthing parent age and year of delivery.

**Results:**

The analysis included 31 241 people with prenatal opioid use (mean [SD] age, 30.0 [5.6] years; 86.1% [26 908 individuals] Canadian-born; 30.6% [9574 individuals] lived in low-income neighborhoods). LCA generated a 5-group model that optimally distinguished opioid users in pregnancy as follows: short-term analgesia with low comorbidity (group 1), analgesia in young people (group 2), medication for opioid use disorder or unregulated opioid use (group 3), pain management with comorbidity (group 4), and mixed opioid use plus high social and medical needs (group 5). The overall risk of postpartum drug overdose or death was 1.5%. Using the 5-group model, compared with people in group 1, the aRR of overdose or death was highest among those in group 5 (aRR, 14.0; 95% CI, 10.1-19.5), followed by group 3 (aRR, 4.6; 95% CI, 3.3-6.5), group 2 (aRR, 3.3; 95% CI, 2.2-4.7), and group 4 (aRR, 3.2; 95% CI, 2.3-4.4).

**Conclusions and Relevance:**

In this cross-sectional study, distinct groups of people with opioid use in pregnancy were associated with increasing degrees of risk of postpartum drug overdose or death. Group characteristics can be used to identify people with high risk and inform harm reduction, home visiting programs, and other interventions.

## Key Points

**Question:**

Are there ways to group people with prenatal opioid use that are associated with postpartum overdose or death?

**Findings:**

In this cross-sectional Canadian study of 31 241 people with prenatal opioid use, a latent class analysis identified 5 distinct groupings: (1) short-term analgesia with low comorbidity, (2) analgesia in young people, (3) medication for opioid use disorder or unregulated opioid use, (4) pain management with comorbidity, and (5) mixed opioid use plus high social and medical needs.

**Meaning:**

These findings suggest that clinically distinct groupings of people with prenatal opioid use can identify those who may benefit from targeted intervention strategies.

## Teaser

This cross-sectional study evaluates the association between the category of opioid use among pregnant people and subsequent overdose or death.

## Introduction

Growth in the opioid overdose crisis has led to opioid use in pregnancy being a public health concern in North America, with 5% of hospital births in Ontario, Canada, exposed to opioids in pregnancy.^1,2^ People use opioids in pregnancy for therapeutic reasons, including pain management and treatment of opioid use disorder (OUD), in addition to unregulated opioids (ie, heroin and fentanyl), with varying risks to the health of pregnant people and infants by type of opioid.^3,4^ Most studies broadly classify opioid exposure in pregnancy on the basis of any prenatal opioid exposure (POE), OUD diagnosis, or infant diagnosis of neonatal abstinence syndrome (NAS).^5,6,7,8,9,10^ Thus, there is limited evidence concerning heterogeneity within these categories, including characterization of diverse circumstances surrounding type of opioid use in pregnancy and indications for opioid use, and the respective outcomes for the health of pregnant people and infants.

Studies have shown that pregnant people with OUD or unregulated opioid use have high rates of social vulnerability and concomitant psychiatric diagnoses and polysubstance use,^5,9,11,12^ whereas characteristics associated with opioid analgesic use in pregnancy include medical comorbidity with fewer socioeconomic disparities.^2,8,13^ However, it may be difficult to identify higher risk groups in studies where POE is defined broadly. For example, although most people with POE are prescribed short courses of opioid analgesics in pregnancy,^2^ those with long-term opioid analgesic use are at greater risk of developing OUD,^14^ which may go undiagnosed because of stigma associated with drug use during pregnancy. Previous studies have identified factors associated with various types of opioid use in pregnancy using variable-centered approaches^5,11,13^ (eg, regression analysis) that focus on associations between variables across people with an underlying assumption that variable associations are the same among all members in a population.^15^ We aimed to provide a different, more holistic perspective using latent class analysis (LCA), a person-centered approach, to identify patterns of variables within people. This approach assumes the interplay of variables varies for different people.^15^ Person-centered approaches are commonly used to identify higher risk groups and inform tailored interventions.^16,17^ For example, LCA was recently used to identify subgroups of medically complex patients and develop 7 tailored treatment strategies.^18^

Comprehensive data on the interplay of multiple sociodemographic and medical characteristics by type of opioid used in pregnancy are lacking. This exploratory study addresses knowledge gaps by conducting an LCA, including several characteristics from population-based linked health records in a province with universal health care, to identify pregnant people with high risk who may benefit from targeted intervention strategies. The objectives were to (1) develop clinically distinguishable groups of people who use opioids in pregnancy and (2) evaluate their association with adverse outcomes (drug overdose or all-cause mortality) within 365 days after the index birth hospitalization.

## Methods

### Study, Setting, and Participants

This population-based repeated cross-sectional study followed the Strengthening the Reporting of Observational Studies in Epidemiology (STROBE) and Reporting of Studies Conducted Using Observational Routinely Collected Data (RECORD) reporting guidelines.^19,20^ We used linked health and demographic administrative databases at ICES (formerly the Institute for Clinical Evaluative Sciences), an independent, nonprofit research institute whose legal status under Ontario’s health information privacy law allows it to collect and analyze health care and demographic data, without consent, for health system evaluation and improvement. We identified births in a data set of birthing parent delivery and newborn records linked to prescription opioid data, emergency department visits, hospitalizations, physician visits, census data, a population registry, and immigration data using unique encoded identifiers and analyzed at ICES. Study data sets are detailed in eTable 1 in the Supplement and described elsewhere.^21,22,23,24^ This study was approved by the University of Toronto and the Hospital for Sick Children research ethics boards.

The study population comprised people aged 12 to 50 years who had a live birth or stillbirth between January 1, 2014, and December 31, 2019, with prenatal opioid use, as described below.^25^ We included people eligible for Ontario’s universal publicly funded health care insurance program during pregnancy and 2 years before conception. One birth per person was randomly selected for those with more than 1 birth in the study period.

### Variables and Outcomes

Opioid use in pregnancy was ascertained through prenatal prescription opioid data, outpatient visits for opioid agonist therapy (OAT), opioid-related hospital records, and/or newborn hospital records for NAS.^25^ Opioid use in pregnancy was classified into mutually exclusive categories, (1) less than 30 days, (2) 30 to 89 days, or (3) 90 or more days of cumulative opioid analgesic use; (4) only methadone OAT; (5) only buprenorphine OAT; (6) other OAT (methadone and buprenorphine, or unspecified OAT); (7) OAT and opioid analgesic; (8) other opioid exposure on the basis of NAS diagnoses and no opioid dispensing records in pregnancy; and (9) opioid-related hospital care, including OUD, poisoning, or adverse events. Pregnant people with opioid use in categories 8 and 9 who did not have prescription opioid records or outpatient OAT were presumed to be using unregulated opioids (ie, heroin or fentanyl).

We included a range of sociodemographic variables including neighborhood-level low-income quintile,^26^ rural residence, number of previous live births, immigrant status,^24^ and social vulnerability (eg, violence-related hospital care, homelessness recorded in health records,^27^ or involvement in criminal justice system recorded in health records). Measures of antecedent care and comorbidities included mental health–related health care, nonopioid substance use-related health care,^28^ pain-related health care composite (eg, low back/abdominal, migraine/headache, chronic pain conditions, or cancer),^29,30^ as well as other chronic conditions from validated registries (eg, rheumatoid arthritis,^31^ diabetes,^32,33^ hypertension,^33,34^ or asthma^35^), and a description of OAT initiation during pregnancy. High medical comorbidity was measured using Johns Hopkins ACG System Aggregated Diagnosis Groups version 10. All variables are detailed in eTable 1 in the Supplement.

The primary outcome was nonfatal and fatal drug overdose or all-cause mortality within 365 days after the index birth hospitalization,^36^ ascertained through hospital records and population registry (eTable 1 in the Supplement). Nonfatal and fatal drug overdose and all-cause mortality were evaluated separately as secondary outcomes.

### Statistical Analysis

An LCA was performed to identify clinically distinct groups using RStudio and poLCA statistical software version 0.98.1091 (R Project for Statistical Computing).^37^ LCA is a finite mixture model that identifies unique groups (latent classes) on the basis of similar responses to variables of interest.^37^ Opioid use in pregnancy and all characteristics of pregnant people were included in the LCA. We examined models with 2 to 9 groups. Group membership was determined using a modal approach, a commonly used method that assigns the most likely group according to the highest membership probability.^38^ Model fit was evaluated on the basis of statistical fit and model interpretability (eTable 2 in the Supplement).

To investigate whether the groups were differentially associated with postpartum drug overdose or all-cause mortality, modified Poisson with robust error variance was used to estimate crude and adjusted relative risks (aRRs) and 95% CIs.^39^ To address our second objective, a subcohort of people with births from January 1, 2014, to June 30, 2019, was identified to allow for 365 days of follow-up. The referent group was the largest group characterized by short-term analgesia with low comorbidity. An offset term was included to account for varying follow-up time. Adjusted analyses included year of delivery and birthing parent age in years at index birth to account for temporal changes in opioid use and residual confounding associated with birthing parent age. The purpose of the analysis was to determine whether the groups were differentiated by risk of the outcome; as such, we did not adjust for additional variables. Multivariable logistic regression was used to compute the C statistic to assess model discrimination. Finally, to further understand the utility of LCA, we performed an additional Poisson regression analysis with type of opioid use in pregnancy as the exposure. Regression analyses were performed using SAS Enterprise Guide statistical software version 7.15 (SAS Institute). Data were analyzed from August 2020 to January 2021.

Missing neighborhood-level income data reflects neighborhoods with high residential instability and are typically low-income and urban and were recoded to quintile 1. Missing residential location was recoded to urban. All other data were complete.

## Results

There were 698 458 hospital births, of which 33 977 (4.9%) had POE. One birth per person was randomly selected, resulting in an analytical sample of 31 241 people for the LCA (Table 1). The mean (SD) age of people who used opioids in pregnancy was 30.0 (5.6) years, all participants (31 241 individuals) were female, 86.1% (26 908 individuals) were Canadian born or long-term residents of Canada, 30.6% (9574 individuals) lived in low-income neighborhoods, and 23.8% (7436 individuals) had pain-related hospital care 2 years before conception. Most opioid use in pregnancy was short-term (<30 days) opioid analgesics (21 965 individuals [70.3%]).

**Table 1.** Characteristics of People Who Used an Opioid During Pregnancy in Ontario, 2014-2019

Characteristic	Patients, No. (%) (N = 31 241 )
Opioid use in the index pregnancy	
Analgesic prescribed for <30 d	21 965 (70.3)
Analgesic prescribed for 30-89 d	1541 (4.9)
Analgesic prescribed for ≥90 days	1329 (4.3)
Methadone OAT only	2560 (8.2)
Buprenorphine OAT only	861 (2.8)
Other OAT	359 (1.1)
OAT plus prescribed analgesic	485 (1.6)
Neonatal abstinence syndrome alone	1686 (5.4)
Opioid-related hospital care only	455 (1.5)
OAT initiation during pregnancy	723 (2.3)
Demographics at the index pregnancy conception	
Age during index pregnancy, y	
12-19	811 (2.6)
20-24	4730 (15.1)
25-29	8642 (27.7)
30-34	10 010 (32.0)
35-39	5623 (18.0)
40-50	1425 (4.6)
Neighborhood level income quintile	
1 (lowest or unknown)	9574 (30.6)
5 (highest)	3908 (12.5)
Rural residence	4539 (14.5)
Immigrant or recent Ontario Health Insurance Plan registrant	4333 (13.9)
≥3 prior live births	3973 (12.7)
Social vulnerability within 2 y preceding the index pregnancy conception	
Social vulnerability composite	2304 (7.4)
Infant discharged to social services at birth	955 (3.1)
Criminal justice system involvement^a^	725 (2.3)
Homelessness^a^	261 (0.8)
Violence-related health care use	848 (2.7)
Medical morbidity within 2 y preceding the index pregnancy conception^b^	
Mental health–related emergency department visit or hospitalization	2351 (7.5)
Nonopioid or multidrug-related health care	1101 (3.5)
Alcohol-related health care	1039 (3.3)
Tobacco-related health care	1098 (3.5)
HIV or hepatitis at any prior time	535 (1.7)
Pain-related hospital care^c^	7436 (23.8)
High medical comorbidity	7820 (25.0)
Asthma within 5 y before conception	597 (1.9)
Chronic obstructive pulmonary disease at any prior time	268 (0.9)
Obesity	1403 (4.5)
Chronic hypertension at any prior time	1227 (3.9)
Diabetes at any prior time	1485 (4.8)

Abbreviation: OAT, opioid agonist therapy.

^a^ As noted within health care records, which may not be comprehensive.

^b^ Unless otherwise specified.

^c^ Pain conditions include low back pain/abdominal pain, migraine, cancer and chronic pain conditions (rheumatoid arthritis, fibromyalgia, joint pain, chronic pancreatitis, peripheral neuropathy, sickle cell disease, and kidney calculi).

### Description of the Latent Groups

Model fit statistics for the LCA and estimated probabilities of group membership for models with 2 to 9 groups are shown in eTable 2 in the Supplement. No model had superior statistical fit. Models with 6 to 9 groups had small group sizes. Model interpretability was best with 3- to 5-group models. Characteristics of the 3- and 4-group models are provided in eTable 3 and eTable 4 in the Supplement. The 5-group model solution is interpreted below. Groups were labeled according to key defining characteristics.

In the 5-group model, class 1 (short-term analgesia with low comorbidity; 13 831 individuals [44%]) was the largest group (Table 2). This group had the highest percentage of short-term opioid analgesic use in pregnancy (<30 days, 12 797 individuals [92.5%]) and lowest rates of economic and social vulnerability and medical morbidity. Group 4 (pain management with comorbidity, 7082 individuals [23%]) had the highest percentage of people with chronic opioid analgesic use (30-89 days; 810 individuals [11.4%], ≥90 days; 990 individuals [14.0%]) and high medical comorbidity (4779 individuals [67.5%]). Group 2 (analgesia in young people, 4601 individuals [15%]) was characterized by short-term analgesic use (3828 individuals [83.2%]) and had the highest representation of people aged 12 to 19 years (637 individuals [13.8%]) and 20 to 24 years (2619 individuals [56.9%]). More than one-third (1673 individuals [36.4%]) of people in this group lived in low-income areas, with 2197 individuals (47.8%) receiving pain-related hospital care and 751 individuals (16.3%) receiving mental health-related hospital care. Group 3 (medication for OUD or unregulated opioid use, 3998 individuals [13%]) was characterized by OAT use (3010 individuals [75.3%]) and unregulated opioid use (928 individuals [23.2%]). This group had the largest percentage of people aged 25 to 29 years (1536 individuals [38.4%]), residing in rural (1237 individuals [30.9%]) and low-income (2185 individuals [54.7%]) areas with high parity (1073 individuals [26.8%]). Group 5 (mixed opioid use plus high social and medical needs; 1729 individuals [6%]) was characterized by OAT (829 individuals [47.9%]), unregulated opioid use (453 individuals [26.2%]) and short-term opioid analgesic use (367 individuals [21.2%]). This group had the highest percentage of social vulnerability (859 individuals [49.7%]), mental health hospital care (956 individuals [55.3%]), high medical comorbidity (1273 individuals [73.6%]), polysubstance use hospital care (nonopioid, 895 individuals [51.8%]; alcohol, 654 individuals [37.8%]), and people living in low-income neighborhoods (850 individuals [49.2%]).

**Table 2.** Characteristics of People Who Used an Opioid During Pregnancy Stratified by the 5-Group Solution Identified by the Latent Class Analyses, 2014 to 2019

Characteristic	Patients, No. (%) (N = 31 241 )^a^				
	Group 1: short-term analgesia with low comorbidity (n = 13 831)	Group 2: analgesia in young people (n = 4601)	Group 3: medication for OUD or unregulated opioid use (n = 3998)	Group 4: pain management with comorbidity (n = 7082)	Group 5: mixed opioid use plus high social and medical needs (n = 1729)
Opioid use in the index pregnancy					
Analgesic prescribed for <30 d	12 797 (92.5)	3828 (83.2)	16 (0.4)	4957 (70.0)	367 (21.2)
Analgesic prescribed for 30-89 d	551 (4.0)	122 (2.7)	17 (0.4)	810 (11.4)	41 (2.4)
Analgesic prescribed for ≥90 days	259 (1.9)	14 (0.3)	27 (0.7)	990 (14.0)	39 (2.3)
Methadone OAT only	21 (0.2)	119 (2.6)	1866 (46.7)	102 (1.4)	452 (26.1)
Buprenorphine OAT only	Suppressed	Suppressed	650 (16.3)	68 (1.0)	132 (7.6)
Other OAT	0	Suppressed	276 (6.9)	Suppressed	80 (4.6)
OAT plus prescribed analgesic	0	17 (0.4)	218 (5.5)	85 (1.2)	165 (9.5)
Neonatal abstinence syndrome alone	192 (1.4)	475 (10.3)	603 (15.1)	62 (0.9)	354 (20.5)
Opioid-related hospital care only	Suppressed	18 (0.4)	325 (8.1)	Suppressed	99 (5.7)
OAT initiation during pregnancy	0	0	550 (13.8)	0	173 (10.0)
Proportion of days covered by opioid use in pregnancy, median (IQR), %^b^	1.5 (1.0-2.6)	1.5 (1.1-2.9)	83.1 (36.9-96.5)	2.7 (1.1-14.2)	38.9 (2.7-88.7)
Demographics at the index pregnancy conception					
Age in index pregnancy, y					
12-19	0	637 (13.8)	57 (1.4)	0	117 (6.8)
20-24	729 (5.3)	2619 (56.9)	775 (19.4)	116 (1.6)	491 (28.4)
25-29	3715 (26.9)	1309 (28.5)	1536 (38.4)	1545 (21.8)	537 (31.1)
30-34	5590 (40.4)	36 (0.8)	1121 (28.0)	2890 (40.8)	373 (21.6)
35-39	3108 (22.5)	0	440 (11.0)	1908 (26.9)	167 (9.7)
40-50	689 (5.0)	0	69 (1.7)	623 (8.8)	44 (2.5)
Neighborhood level income quintile					
1 (lowest or unknown)	2785 (20.1)	1673 (36.4)	2185 (54.7)	2081 (29.4)	850 (49.2)
5 (highest)	2360 (17.1)	352 (7.7)	223 (5.6)	835 (11.8)	138 (8.0)
Rural residence	1457 (10.5)	897 (19.5)	1237 (30.9)	617 (8.7)	331 (19.1)
Immigrant or recent Ontario Health Insurance Plan registrant	2732 (19.8)	92 (2.0)	14 (0.4)	1460 (20.6)	35 (2.0)
≥3 prior live births	1256 (9.1)	32 (0.7)	1073 (26.8)	1295 (18.3)	317 (18.3)
Social vulnerability within 2 y preceding index pregnancy conception					
Social vulnerability composite	147 (1.1)	275 (6.0)	764 (19.1)	259 (3.7)	859 (49.7)
Infant discharged to social services at birth	40 (0.3)	74 (1.6)	434 (10.9)	60 (0.8)	347 (20.1)
Criminal justice system involvement^c^	78 (0.6)	42 (0.9)	218 (5.5)	98 (1.4)	289 (16.7)
Homelessness^c^	Suppressed	18 (0.4)	36 (0.9)	Suppressed	193 (11.2)
Violence-related health care use	27 (0.2)	157 (3.4)	193 (4.8)	101 (1.4)	370 (21.4)
Medical morbidity within 2 y preceding index pregnancy conception^d^					
Mental health–related emergency department visit or hospitalization	26 (0.2)	751 (16.3)	179 (4.5)	439 (6.2)	956 (55.3)
Anxiety	17 (0.1)	473 (10.3)	97 (2.4)	298 (4.2)	600 (34.7)
Mood disorders	7 (0.1)	318 (6.9)	44 (1.1)	153 (2.2)	327 (18.9)
Self-harm	Suppressed	78 (1.7)	40 (1.0)	Suppressed	295 (17.1)
Schizophrenia	0	17 (0.4)	10 (0.3)	11 (0.2)	63 (3.6)
Nonopioid/multidrug-related health care	0	57 (1.2)	142 (3.6)	7 (0.1)	895 (51.8)
Alcohol-related health care	39 (0.3)	138 (3.0)	101 (2.5)	107 (1.5)	654 (37.8)
Tobacco-related health care	103 (0.7)	217 (4.7)	219 (5.5)	316 (4.5)	243 (14.1)
HIV or hepatitis at any prior time	47 (0.3)	9 (0.2)	164 (4.1)	114 (1.6)	201 (11.6)
Pain-related hospital care	548 (4.0)	2197 (47.8)	343 (8.6)	3526 (49.8)	822 (47.5)
Chronic pain conditions^e^	407 (2.9)	1911 (41.5)	298 (7.5)	2822 (39.8)	727 (42.0)
Migraine	340 (2.5)	42 (0.9)	52 (1.3)	721 (10.2)	72 (4.2)
Low back or abdominal pain	74 (0.5)	280 (6.1)	31 (0.8)	570 (8.0)	98 (5.7)
Cancer	10 (0.1)	8 (0.2)	Suppressed	43 (0.6)	Suppressed
High medical comorbidity	Suppressed	1573 (34.2)	Suppressed	4779 (67.5)	1273 (73.6)
Asthma within 5 y before conception	115 (0.8)	149 (3.2)	25 (0.6)	236 (3.3)	72 (4.2)
Chronic obstructive pulonary disease at any prior time	68 (0.5)	0	19 (0.5)	147 (2.1)	34 (2.0)
Obesity	381 (2.8)	92 (2.0)	23 (0.6)	860 (12.1)	47 (2.7)
Chronic hypertension at any prior time	340 (2.5)	42 (0.9)	52 (1.3)	721 (10.2)	72 (4.2)
Diabetes at any prior time	345 (2.5)	71 (1.5)	106 (2.7)	880 (12.4)	83 (4.8)

Abbreviations: OAT, opioid agonist therapy; OUD, opioid use disorder.

^a^ Sample sizes with fewer than 6 participants were suppressed.

^b^ Based on people with prescription opioid records: group 1 (13 632 individuals), group 2 (4108 individuals), group 3 (2872 individuals), group 4 (7013 individuals), and group 5 (1240 individuals). Proportion of days covered with opioid use in pregnancy is calculated as follows: (total days covered with opioid use in pregnancy / length of pregnancy in days) × 100.

^c^ As noted within health care records, which may not be comprehensive.

^d^ Unless otherwise specified.

^e^ Chronic pain conditions include rheumatoid arthritis, fibromyalgia, joint pain, chronic pancreatitis, peripheral neuropathy, sickle cell disease, and kidney calculi.

### Risk of Postpartum Drug Overdose or All-Cause Mortality

Of the 28 983 people who gave birth between January 1, 2014, and June 30, 2019, 427 (1.5%) experienced a drug overdose or death within 365 days after the index birth hospitalization, ranging from 61 (0.5%) in the short-term analgesia with low comorbidity group to 111 (7.0%) in the mixed opioid use plus high social and medical needs group. The aRRs were significantly higher for all latent groups compared with the short-term analgesia with low comorbidity group (eFigure in the Supplement). In the 5-group model, the greatest risk was among patients with mixed opioid use plus high social and medical needs (aRR, 14.0; 95% CI, 10.1-19.5), followed by those taking medication for OUD or unregulated opioid use (aRR, 4.6; 95% CI, 3.3-6.5), analgesia in young people (aRR, 3.3; 95% CI, 2.2-4.7), and pain management with comorbidity (aRR, 3.2; 95% CI, 2.3-4.4) (Figure). The C statistic was 0.73 (95% CI, 0.71-0.75). Secondary analyses of each outcome separately included 392 (1.4%) drug overdoses and 46 deaths (0.2%). Associations by latent group were similar for drug overdose and all-cause mortality separately (eTable 5 in the Supplement).

**Figure.**
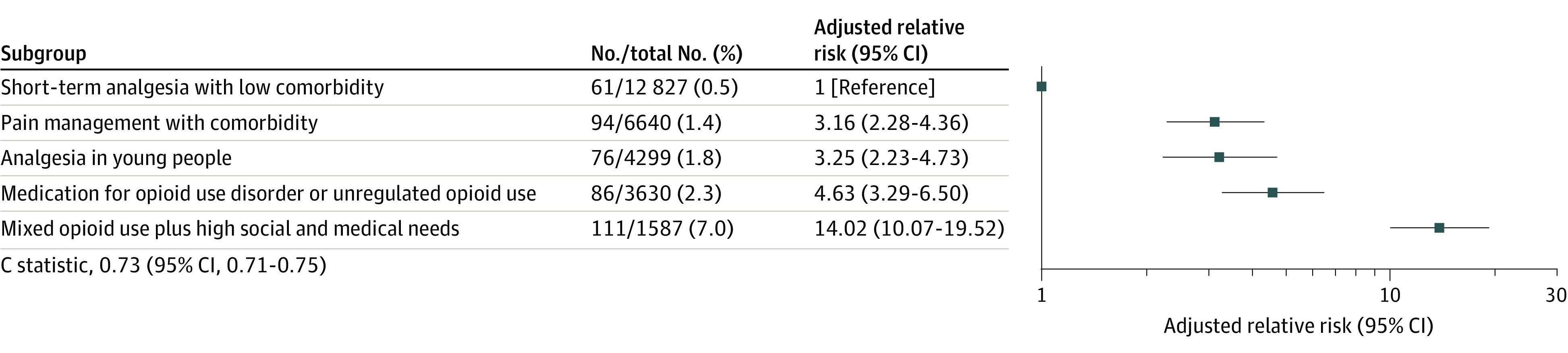
Risk of Drug Overdose or Death up to 365 Days After the Index Birth Hospitalization, Presented According to the Groups Identified in the Optimal 5-Group Latent Class Analysis Solution Included are 28 983 people with a birth between January 1, 2014, and June 30, 2019. Relative risks are adjusted for birthing parent age and year of delivery. The C statistic was computed using multivariable logistic regression.

When the exposure was redefined as type of POE, compared with people with less than 30 days of opioid analgesic use, people with both OAT and opioid analgesic use had the highest risk of drug overdose or death within 365 days after the index birth hospitalization (aRR, 5.4; 95% CI, 3.5-8.4) (Table 3). Risk of the outcome was similar between people with unregulated opioid use (aRR, 3.4; 95% CI, 2.6-4.5) and OAT (aRR, 3.0; 95% CI, 2.3-3.8). Risk increased with duration of opioid analgesic use (30-89 days, aRR, 1.6; 95% CI, 1.0-2.6; ≥90 days, aRR, 2.6; 95% CI, 1.7-3.9) compared with people with less than 30 days of opioid analgesic use. None of the aRRs by type of POE approached that of the highest risk group, mixed opioid use plus high social and medical needs, identified in the 5-group LCA.

**Table 3.** Risk of Experiencing Drug Overdose and All-Cause Mortality Within 365 Days After the Index Birth Hospitalization by Type of Prenatal Opioid Exposure and LCA 5-Group Solution

Variable	Patients at risk, No.	Events, No.	Patients with outcome, %	aRR (95% CI)^a^	C statistic (95% CI)
5-Group LCA model					0.73 (0.71-0.75)
Short-term analgesia with low comorbidity (group 1)	12 827	61	0.5	1 [Reference]	
Analgesia in young people (group 2)	4299	76	1.8	3.25 (2.23-4.73)	
Medication for opioid use disorder or unregulated opioid use (group 3)	3630	85	2.3	4.63 (3.29-6.50)	
Pain management with comorbidity (group 4)	6640	94	1.4	3.16 (2.28-4.36)	
Mixed opioid use plus high social and medical needs (group 5)	1587	111	7.0	14.02 (10.07-19.52)	
Type of prenatal opioid exposure					0.69 (0.66-0.72)
Opioid analgesic <30 d	20 474	187	0.9	1 [Reference]	
Opioid analgesic 30-89 d	1434	20	1.4	1.61 (1.02-2.55)	
Opioid analgesic ≥90 d	1236	26	2.1	2.61 (1.73-3.94)	
OAT (methadone and/or buprenorphine)	3426	101	2.9	2.97 (2.32-3.80)	
OAT plus analgesic	447	22	4.9	5.39 (3.46-8.41)	
Unregulated opioid use	1966	71	3.6	3.39 (2.55-4.51)	

Abbreviations: aRR, adjusted relative risk; LCA, latent class analysis; OAT, opioid agonist therapy.

^a^ Relative risks are adjusted for birthing parent age and year of delivery.

## Discussion

In this large population-based, cross-sectional study, we included multiple factors from linked health records to identify distinct groups among people with opioid use in pregnancy. In the 5-group model, these included (1) short-term analgesia with low comorbidity, (2) analgesia in young people, (3) medication for OUD or unregulated opioid use, (4) pain management with comorbidity, and (5) mixed opioid use plus high social and medical needs. All groups had varying risk profiles and distinct outcomes profiles. Although the absolute risk of drug overdose or death in the 365 days after the index birth hospitalization was low overall (1.5%), there were elevated risks among all latent groups compared with people with short-term analgesia with low comorbidity. The highest risk among the smallest group was characterized by mixed opioid use plus high social and medical needs. When the exposure was redefined as type of POE, the highest outcome risk was among people with both OAT and opioid analgesic use, followed by unregulated opioid use, OAT, and long-term opioid analgesic use. The LCA demonstrated that the inclusion of sociodemographic and medical characteristics was useful to identify groupings with a wider range of risk, especially the highest risk group of mixed opioid use plus high social and medical needs, whereas redefining the exposure to type of POE identified people with both OAT and opioid analgesic use as having the highest outcome risk. Taken together, both methods are clinically useful to identify people with high risk and demonstrate the importance of examining POE in more depth.

To our knowledge, this is the first study to apply a person-centered approach to identify distinct groups of people while accounting for multiple types of opioids in a single study. Our findings build on previous studies^8,10,13^ showing that people with prenatal opioid analgesic use were more likely than unexposed people to have medical comorbidity, pain diagnoses, psychiatric comorbidities, and polysubstance use. In our study, people with prenatal opioid analgesic use represented a heterogeneous group that was divided into 4 groups. The group characterized by mixed opioid use (including opioid analgesics) plus high social and medical needs were most likely to have hospital care for mental health, polysubstance use, and high social vulnerability. We also identified a younger group with short-term use of opioids for pain management with complex needs, including high rates of hospital care associated with pain and mental health. Pain diagnoses and high medical comorbidity were highest among groups with pain management with comorbidity, mixed opioid use plus high social and medical needs, and analgesia in young people. The largest group identified, short-term analgesia with low comorbidity, had the lowest risk profile with no medical morbidity. These unique groups highlight the importance of providing treatment to address varying complex needs and engaging pregnant people at young ages to prevent adverse health outcomes.

Previous studies^5,9,11,12,40,41,42^ have reported associations between people with any opioid use or OUD during pregnancy and economic disadvantage, social vulnerability, higher psychiatric comorbidities, polysubstance use, and chronic medical conditions. In this study, the group characterized by medication for OUD or unregulated opioid use had high rates of social and economic disadvantage; however, the highest rates of social vulnerability, hospital care for mental health and polysubstance use, HIV or hepatitis, and high medical comorbidity were among the group with mixed opioid use plus high social and medical needs.

The LCA grouped people receiving OAT with people using unregulated opioids in pregnancy, suggesting that these groups share similar attributes despite being clinically differentiated by treatment for OUD. In additional analyses by type of POE, we found similar risks of postpartum drug overdose or death between people receiving OAT and those with unregulated opioid use. This finding was unexpected as previous studies^43,44^ have reported a reduction in the risk of overdose or death with OAT outside of pregnancy. Because pregnancy is often a transition time from unregulated opioid use to OAT, we hypothesize that some people may have used both OAT and unregulated opioids during pregnancy or had low treatment adherence.^45^

Pregnancy is an opportune time to identify OUD and facilitate coordination of comprehensive care, particularly for people with infrequent interactions with the health care system.^46,47^ Our findings provide a detailed description of the complex social and clinical needs associated with different types of prenatal opioid use and can support practitioners or institutions in identifying high-risk people and inform the development of harm reduction strategies and tailored comprehensive care. The groups identified in the LCA and guidelines published by the American College of Obstetrics and Gynecology and The Society of Obstetricians and Gynaecologists of Canada could be used to identify potential strategies, including ensuring that conservative prescribing guidelines are followed to prevent OUD, using nonopioid pain management strategies, screening for substance misuse, substance use treatment (including low-barrier access to OAT during pregnancy through to the postpartum period), psychiatric treatment, and social support services.^46,48^ It is important to note that people who use substances in pregnancy are often stigmatized and face numerous barriers to care, including fear of child apprehension and judgment from practitioners.^46^ Establishing a patient-practitioner rapport that is flexible, nonjudgmental and empathetic, establishing confidential guidelines of care, and facilitating family-centered comprehensive care are imperative strategies to incorporate into public health programming and clinical care.^47,49^ For example, a recent mixed-methods evaluation of 8 comprehensive care programs across Canada serving pregnant and parenting people with substance use reported positive client experiences (eg, feeling safe or trusting staff) and outcomes (eg, strengthened connection to children and communities, safer housing, and improved health). Notably, these programs were grounded in best practice approaches (eg, trauma-informed and culturally grounded) and program staff delivered care using a nonjudgmental approach, which was reported to be a key factor underlying safe and trusting relationships with clients that led to more positive health and social outcomes.^50^

We explored the granularity available with this analysis, recognizing that this study did not identify a statistically optimal model and that model selection will vary with purpose and resources. For example, the 5-group model identified different groupings, some with complex needs relevant for targeted interventions. To support development of broader interventions, groups with similar needs could be combined, as in the 3-group and 4-group solutions. Findings can be used to inform future studies to evaluate the impact of multiple risk factors and opioid use on the health of pregnant people and children, and account for confounding by indication. Variables were identified using diagnostic codes, prescriptions, and routinely collected information and can be replicated in other jurisdictions with health administrative data.

### Limitations

There are limitations inherent with health administrative data, which reflect people connected to the health care system. Thus, we may have missed births with POE; however, this outcome is likely minimal, as 98% of births in Ontario occur in hospitals.^51^ Certain constructs may be underreported (ie, homelessness,^27^ unregulated opioid use, polysubstance use, or nonfatal overdoses not resulting in hospital care) or misclassified by inconsistent diagnostic coding. We mitigated this risk using validated and previously used measures and multiple linked databases. We had access to area-level income, which has low agreement with individual-level income, although it is still associated with similar socioeconomic gradients in health outcomes.^52,53^ This suggests that area-level income measures capture unique contributing contextual information related to socioeconomic position that is important to understand population-level health inequities. We took an as-prescribed approach to identify prescription opioid use, which assumes that people took the full course of dispensed opioids. This may result in exposure misclassification and overestimation of prenatal opioid use. Groups were not validated against a reference standard; however, we examined external validity with an outcome analysis.

## Conclusions

Our findings highlight the heterogeneity among people who use opioids in pregnancy and the utility of LCA to identify meaningful groups with distinct risk profiles and health care needs. Findings from this study can inform strategies to prevent opioid use in pregnancy and reduce opioid-related harm. This study has important implications for early identification of high-risk people and the development of evidence-based tailored comprehensive care to support health outcomes for people affected by opioid use in pregnancy.

## Notes

Supplement. **eTable 1.** Methods Used in the Current Study to Describe Opioid Use in Pregnancy and Other Variables **eTable 2.** Indicators of Model Fit for Each Latent Class Analysis, Among 31 241 People Who Used an Opioid During Pregnancy, 2014 to 2019 **eTable 3.** Characteristics of 31 241 People Who Used an Opioid During Pregnancy Stratified by the 3-Group Solution Identified by the Latent Class Analyses, 2014 to 2019 **eTable 4.** Characteristics of 31 241 People Who Used an Opioid During Pregnancy Stratified by the 4-Group Solution Identified by the Latent Class Analyses, 2014 to 2019 **eTable 5.** Risk of Experiencing Drug Overdose and All-Cause Mortality Within 365 Days After the Index Birth Hospitalization, Shown by the 3, 4 and 5 Group Solutions Identified by the Latent Class Analyses **eFigure.** Risk of Drug Overdose/Death 365 Days After Birth Hospitalization by Groups Identified by LCA
