# Error in Text, Tables, Figure, and Supplement

**DOI:** 10.1001/jamanetworkopen.2022.23058

**Published:** 2022-07-07

In the Original Investigation titled “Categorization of Opioid Use Among Pregnant People and Association With Overdose or Death,”^1^ published May 27, 2022, there was an error in the text, tables, figure, and supplement. These sections should have stated that adjusted rate ratios were calculated, not adjusted relative risks. This article has been corrected.^1^
