# Supplementary material for: Categorization of Opioid Use Among Pregnant People and Association With Overdose or Death
Source: JAMA Netw Open. 2022 May 27;5(5):e2214688. doi: 10.1001/jamanetworkopen.2022.14688 (PMC9142862; doi:10.1001/jamanetworkopen.2022.14688)
Supplement: Supplement. — eTable 1. Methods Used in the Current Study to Describe Opioid Use in Pregnancy and Other Variables eTable 2. Indicators of Model Fit for Each Latent Class Analysis, Among 31 241 People Who Used an Opioid During Pregnancy, 2014 to 2019 eTable 3. Characteristics of 31 241 People Who Used an Opioid During Pregnancy Stratified by the 3-Group Solution Identified by the Latent Class Analyses, 2014 to 2019 eTable 4. Characteristics of 31 241 People Who Used an Opioid During Pregnancy Stratified by the 4-Group Solution Identified by the Latent Class Analyses, 2014 to 2019 eTable 5. Risk of Experiencing Drug Overdose and All-Cause Mortality Within 365 Days After the Index Birth Hospitalization, Shown by the 3, 4 and 5 Group Solutions Identified by the Latent Class Analyses eFigure. Risk of Drug Overdose/Death 365 Days After Birth Hospitalization by Groups Identified by LCA [file jamanetwopen-e2214688-s001.pdf]

# Supplemental Online Content

Camden A, To T, Ray JG, Gomes T, Bai L, Guttmann A. Categorization of opioid use among pregnant people and association with overdose or death. *JAMA Netw Open*. 2022;5(5):e2214688. doi:10.1001/jamanetworkopen.2022.14688

**eTable 1.** Methods Used in the Current Study to Describe Opioid Use in Pregnancy and Other Variables

**eTable 2.** Indicators of Model Fit for Each Latent Class Analysis, Among 31 241 People Who Used an Opioid During Pregnancy, 2014 to 2019

**eTable 3.** Characteristics of 31 241 People Who Used an Opioid During Pregnancy Stratified by the 3-Group Solution Identified by the Latent Class Analyses, 2014 to 2019

**eTable 4.** Characteristics of 31 241 People Who Used an Opioid During Pregnancy Stratified by the 4-Group Solution Identified by the Latent Class Analyses, 2014 to 2019

**eTable 5.** Risk of Experiencing Drug Overdose and All-Cause Mortality Within 365 Days After the Index Birth Hospitalization, Shown by the 3, 4 and 5 Group Solutions Identified by the Latent Class Analyses

**eFigure.** Risk of Drug Overdose/Death 365 Days After Birth Hospitalization by Groups Identified by LCA

This supplemental material has been provided by the authors to give readers additional information about their work.

© 2022 Camden A et al. *JAMA Network Open.*

## eTable 1. Methods Used in the Current Study to Describe Opioid Use in Pregnancy and Other Variables

| Measure | Definition | Timing | Data source(s) |
|---|---|---|---|
| Opioid use in pregnancy | (1) less than 30 days of cumulative opioid analgesic use<br>(2) 30-89 days of cumulative opioid analgesic use<br>(3) 90+ days of cumulative opioid analgesic use<br>(4) methadone for OAT<br>(5) buprenorphine for OAT<br>(6) OAT – other (methadone and buprenorphine use or unspecified OAT use)<br>(7) OAT and opioid analgesic use<br>(8) other opioid exposure – neonatal abstinence syndrome only (ICD-10 P96.1 OR ICD-10 P04.4)<br>(9) other opioid exposure – gestational parent opioid-related hospital care (ICD-10 opioid use disorder: F11, DSM-5 304.00, 305.50; opioid poisoning T40.0-T40.4, T40.6; adverse effects of opioids Y45.0)<br><br>Prescription opioid analgesics for pain included: morphine, codeine, oxycodone, hydrocodone, hydromorphone, meperidine, tramadol, methadone, buprenorphine, fentanyl, tapentadol<br><br>Unspecified OAT included OHIP codes: K682 (Opioid agonist maintenance program monthly management fee – intensive); K683 (Opioid agonist maintenance program monthly management fee – maintenance); K684 (Opioid agonist maintenance program monthly management fee - team maintenance) | Conception to delivery; NAS - Newborn birth episode; re-admission within 14 days of life | NMS (https://datadictionary.ices.on.ca/Applications/DataDictionary/Library.aspx?Library=NMS)<br>DAD (https://datadictionary.ices.on.ca/Applications/DataDictionary/Library.aspx?Library=DAD),<br>NACRS (https://datadictionary.ices.on.ca/Applications/DataDictionary/Library.aspx?Library=NACRS),<br>OHMRS (https://datadictionary.ices.on.ca/Applications/DataDictionary/Library.aspx?Library=OMHRS)<br>OHIP (https://datadictionary.ices.on.ca/Applications/DataDictionary/Library.aspx?Library=OHIP)<br><br>Validation studies have demonstrated these databases are complete and accurate:<br>Canadian Institute for Health Information, CIHI Data Quality Study of the 2006–2007 Discharge Abstract Database (Ottawa, Ont.: CIHI, 2009).<br>Martin L, Hirdes J, Morris J, Montague P, Rabinowitz T, Fries B. Validating the Mental Health Assessment Protocols (MHAPs) in the Resident Assessment Instrument Mental Health (RAI-MH). J Psychiatr Ment Health Nurs 2009; 16: 646–53.<br>Schull MJ, Azimaee M, Marra M, Cartagena RG, Vermeulen MJ, Ho M, Guttmann A. ICES: Data, Discovery, Better Health. International Journal of Population Data Science. 2019;4(2). |

© 2022 Camden A et al. *JAMA Network Open.*

| Measure | Definition | Timing | Data source(s) |
|---|---|---|---|
| Proportion of days covered with opioid use in pregnancy | Total number of days covered with any opioid use in pregnancy/length of pregnancy in days*100. | Conception to delivery | NMS |
| Drug overdose or death | ICD-10 T36-T50; Y40-Y59; X60-X69, OR All-cause mortality – death – vital status field | 365 days following discharge from the birth hospitalization | DAD, NACRS, RPDB |
| **Gestational parent substance use-related health care** | | | |
| Any tobacco | ICD-10 F17, T65.2, Z72.0, Z71.6, OHIP DXCODE 305 | 2 years before conception | DAD, NACRS, OHIP (https://datadictionary.ices.on.ca/Applications/DataDictionary/Library.aspx?Library=OHIP) |
| Any alcohol | OHIP DXCODE: 291, 303, ICD10CA G62.1, G31.2, G72.1, I42.6, K29.2, K85.2, K70, K86.0, E24.4, F10, X45, X65, Y15, T51, R78.0, Y90, Y91, Z72.1, Z71.4 | 2 years before conception | DAD, NACRS, OHIP |
| Any non-opioid/multi-drug use | ICD-10 F12, T40.7, F13, F14, T40.5, R78.2, F15, T43, F16, T40.8, T40.9, F18, F19, Z72.2, Z71.5, OHIP DXCODE 292, 304 | 2 years before conception | DAD, NACRS, OHIP |
| **Social vulnerability** | | | |
| Homelessness as recorded on health care records | The record meets any following criteria: (1) DAD: ICD-10 Z590 or homeless=Y or postal code=XX; (2) NACRS: ICD-10 Z590 or residence type= Homeless or postal code=XX; (3) OMHRS: usual residence= Homeless (with or without shelter) | 2 years before conception | DAD, NACRS, OMHRS |
| Health care received while involved with the criminal justice system as recorded on health care records | The record meets any following criteria:<br>(1) institution in OHIP with institution type =Correctional Centre.<br>(2) institution number in DAD with institution type =Correctional Centre and referred from law enforcement<br>(3)instfnum in NACRS with institution type=Correctional Centre and referred from a Legal service.<br>(4) Last 4 digits of X65 in OMHRS with institution type = Correctional Centre. | 2 years before conception | OHIP, DAD, NACRS, OMHRS |

© 2022 Camden A et al. *JAMA Network Open.*

| Measure | Definition | Timing | Data source(s) |
|---|---|---|---|
| Child Apprehension at birth | Discharged to infants and children discharged/detained by social services OR social services OR Z622 OR Z762 | Newborn birth episode | DAD |
| Violence-related health care use | ICD-10: X85-X99, Y00-Y09, Y871, T740-T749 | 2 years before conception | NACRS, DAD |
| **Demographics** | | | |
| Age at current delivery | 12-19, 20-24, 25-29, 30-34, 35-39, 40-50 years | Index delivery | MOMBABY |
| 3+ previous livebirths | Number of previous livebirths derived from all mom's records | Index delivery | MOMBABY |
| Neighbourhood-level income quintile | Groups: Quintile 1 (lowest), Quintile 5 (highest), missing data (represents suppressed) were re-coded to Quintile 1.<br>Note: Missing data are suppressed for neighbourhoods + high residential instability, which are most likely to be low income and urban.<br><br>Area-level measures were ascertained from the census and measured at the dissemination area, Canada's smallest level of geography, containing 400-700 people. | Conception | RPDB (https://datadictionary.ices.on.ca/Applications/DataDictionary/Library.aspx?Library=RPDB), PCCF+ |
| Rural residence | <10,000 residents, missing data were re-coded as urban<br>Note: Missing data are suppressed for neighbourhoods + high residential instability, which are most likely to be low income and urban. | Conception | PCCF+ |
| Immigrant to Canada or recent OHIP registrant | Immigrant to Canada or recent OHIP registrant from June 2017-December 2019 | Conception | RPDB, Immigration, Refugees and Citizenship Canada Permanent Resident Database (https://datadictionary.ices.on.ca/Applications/DataDictionary/Library.aspx?Library=CIC)<br>Chiu M, Lebenbaum M, Lam K, et al. Describing the linkages of the immigration, refugees and citizenship Canada permanent resident data and vital statistics death registry to Ontario's administrative health database. BMC medical informatics and decision making. 2016;16(1):135. |
| **Pre-pregnancy morbidity** | | | |

© 2022 Camden A et al. *JAMA Network Open.*

| Measure | Definition | Timing | Data source(s) |
|---|---|---|---|
| High medical comorbidities | Johns Hopkins ADG score of 10+ | 2 years before conception | DAD, NACRS, OHIP |
| Pain-related hospital care | ICD-10: M54.5 (low back pain) or Abdominal pain (R10), G43, G44 (migraine/headache), rheumatoid arthritis (ICES-derived cohort), M79.7 (fibromyalgia), M25.5, M25.50-M25.59, K07.63, K07.69 (joint pain), K86.0, K86.1 (chronic pancreatitis), E10.41, E11.41, E13.41, E14.41 (peripheral neuropathy), D57.1, D57.2, D57.3, D57.8 (sickle cell disease), N20.0, N20.1, N20.2, N20.9, N21.0, N21.1, N21.8, N21.9, N22.0, N22.8 (renal calculus), cancer | 2 years before conception | DAD, NACRS, Ontario Cancer Registry |
| Any mental health hospitalization or emergency department visit | ICD-10 (DAD/NACRS) DX10CODE1= F06-F09, F20-F99 or DX10CODE2-DX10CODE10 = X60-X84, Y10-Y19, Y28 when DX10CODE1 not equal to F06-F99; ICD-9-CM (OMHRS) Any OMHRS (including missing, except for 290.x, 294.x in primary diagnosis). If primary dx missing and provisional=17, exclude; OMHRS prior to 2016/17 Any OMHRS (including missing, except for 290.x, 294.x in primary diagnosis). If primary dx missing and provisional=2, exclude Self-harm, anxiety, mood disorder, schizophrenia | 2 years before conception | DAD, NACRS, OMHRS |
| HIV or hepatitis | ICD10CA: B16, B180, B181, B171, B182) or OHIP DXCODE: 070, 964 | Ever diagnosed | ICES-derived cohort (HIV; https://datadictionary.ices.on.ca/Applications/DataDictionary/Library.aspx?Library=HIV), DAD, OHIP |
| Asthma | | years before conception | ICES-derived cohort (https://datadictionary.ices.on.ca/Applications/DataDictionary/Library.aspx?Library=ASTHMA) |
| Obesity | OHIP DXCODE 278, or ICD-10CA E66 | 2 years before conception | OHIP, DAD |
| Chronic Obstructive Pulmonary Disease | | Ever diagnosed | ICES-derived cohort (https://datadictionary.ices.on.ca/Applications/DataDictionary/Library.aspx?Library=COPD) |

| Measure | Definition | Timing | Data source(s) |
|---|---|---|---|
| Hypertension | | Ever diagnosed | ICES-derived cohort (https://datadictionary.ices.on.ca/Applications/DataDictionary/Library.aspx?Library=HYPER) |
| Diabetes | | Ever diagnosed | ICES-derived cohort (https://datadictionary.ices.on.ca/Applications/DataDictionary/Library.aspx?Library=ODD) |

DAD Discharge Abstract Database; NACRS National Ambulatory Care Reporting System; OHIP Ontario Health Insurance Plan; OMHRS Ontario Mental Health Reporting System; NMS Narcotic Monitoring System; ODB Ontario Drug Benefit; RPDB Registered Persons Database; PCCF+ Postal Code Conversion File.

© 2022 Camden A et al. *JAMA Network Open.*

## eTable 2. Indicators of Model Fit for Each Latent Class Analysis, Among 31 241 People Who Used an Opioid During Pregnancy, 2014 to 2019

| | Indicators of Model Fit | | | | | | | Predicted Probability of Membership | | | | | | | | |
|---|---|---|---|---|---|---|---|---|---|---|---|---|---|---|---|---|
| **No. latent classes evaluated** | **BIC** | **AIC** | **$G^2$** | **Entropy** | **Log Likelihood** | **LMR Test (vs. k-1)** | | **1** | **2** | **3** | **4** | **5** | **6** | **7** | **8** | **9** |
| **1** | 604843 | 604534 | 91592 | NA | | NA | | 1.00 | | | | | | | | |
| **2** | 590051 | 589425 | 76408 | 0.70 | -294638 | NA | | 0.77 | 0.23 | | | | | | | |
| **3** | 584270 | 583326 | 70232 | 0.70 | -291550 | p < 0.001 | | 0.59 | 0.24 | 0.17 | | | | | | |
| **4** | 580853 | 579593 | 66423 | 0.71 | -289645 | p < 0.001 | | 0.50 | 0.08 | 0.29 | 0.13 | | | | | |
| **5** | 578361 | 576783 | 63537 | 0.70 | -288202 | p < 0.001 | | 0.44 | 0.15 | 0.13 | 0.23 | 0.06 | | | | |
| **6** | 577340 | 575445 | 62123 | 0.67 | -287496 | p < 0.001 | | 0.15 | 0.42 | 0.11 | 0.14 | 0.06 | 0.12 | | | |
| **7** | 576549 | 574336 | 60938. | 0.70 | -286903 | p < 0.001 | | 0.12 | 0.06 | 0.02 | 0.15 | 0.10 | 0.12 | 0.43 | | |
| **8** | 575872 | 573342 | 59869 | 0.70 | -286368 | p < 0.001 | | 0.02 | 0.08 | 0.18 | 0.16 | 0.04 | 0.03 | 0.37 | 0.10 | |
| **9** | 575261 | 572414 | 58864 | 0.68 | -285866 | p < 0.001 | | 0.02 | 0.17 | 0.11 | 0.04 | 0.17 | 0.10 | 0.03 | 0.08 | 0.28 |

Statistical criteria included the Akaike Information Criterion (AIC), Bayesian Information Criterion (BIC), likelihood-ratio chi-square test ($G^2$), where lower values indicate better model fit, Entropy, where higher values indicate greater discrimination between classes, and the Lo-Mendell-Rubin test (LMR), where significant *P* values (< 0.05) indicate significantly better model fit vs. k-1 classes.[37] NA not applicable.

© 2022 Camden A et al. *JAMA Network Open.*

**eTable 3. Characteristics of 31 241 People Who Used an Opioid During Pregnancy Stratified by the 3-Group Solution Identified by the Latent Class Analyses, 2014 to 2019**

| | **Latent Class** | | |
|---|---|---|---|
| **Characteristic, Number (%)** | **Class 1: Short-term analgesia with low comorbidity N=18,313** | **Class 2: Pain management with comorbidity N=7478** | **Class 3: medication for OUD or unregulated opioid use N=5450** |
| **Opioid use in the index pregnancy** | | | |
| Analgesic prescribed for < 30 days | 16,081 (87.8) | 5676 (75.9) | 208 (3.8) |
| Analgesic prescribed for 30-89 days | 716 (3.9) | 811 (10.8) | 14 (0.3) |
| Analgesic prescribed for 90+ days | 524 (2.9) | 797 (10.7) | 8 (0.1) |
| Methadone OAT only | 446 (2.4) | 37 (0.5) | 2077 (38.1) |
| Buprenorphine OAT only | 85 (0.5) | 18 (0.2) | 758 (13.9) |
| Other OAT | Suppressed | Suppressed | 358 (6.6) |
| OAT + prescribed analgesic | 14 (0.1) | 66 (0.9) | 405 (7.4) |
| Neonatal abstinence syndrome alone | 394 (2.2) | 72 (1.0) | 1220 (22.4) |
| Opioid-related hospital care only | Suppressed | Suppressed | 402 (7.4) |
| OAT initiation during pregnancy | 0 (0.0) | 0 (0.0) | 723 (13.3) |
| **Demographics at the index pregnancy conception** | | | |
| Age in index pregnancy, y | | | |
| 12-19 | 213 (1.2) | 320 (4.3) | 278 (5.1) |
| 20-24 | 1769 (9.7) | 1532 (20.5) | 1429 (26.2) |
| 25-29 | 4659 (25.4) | 2059 (27.5) | 1924 (35.3) |
| 30-34 | 6851 (37.4) | 1891 (25.3) | 1268 (23.3) |
| 35-39 | 3930 (21.5) | 1224 (16.4) | 469 (8.6) |
| 40-50 | 891 (4.9) | 452 (6.0) | 82 (1.5) |
| Neighbourhood Level Income Quintile | | | |
| Q1 (lowest or unknown) | 3864 (21.1) | 2609 (34.9) | 3101 (56.9) |
| Q5 (highest) | 2933 (16.0) | 697 (9.3) | 278 (5.1) |
| Rural residence | 2007 (11.0) | 930 (12.4) | 1602 (29.4) |
| Immigrant or recent OHIP registrant | 3373 (18.4) | 925 (12.4) | 35 (0.6) |
| 3+ prior livebirths | 1626 (8.9) | 1026 (13.7) | 1321 (24.2) |
| **Social risk factors within 2y preceding index pregnancy conception** | | | |
| Social risk composite | 215 (1.2) | 524 (7.0) | 1565 (28.7) |
| Infant discharged to social services at birth | 56 (0.3) | 123 (1.6) | 776 (14.2) |
| Criminal justice system involvement[a] | 108 (0.6) | 127 (1.7) | 490 (9.0) |
| Homelessness[a] | Suppressed | Suppressed | 203 (3.7) |
| Violence-related health care use | 52 (0.3) | 287 (3.8) | 509 (9.3) |
| **Medical morbidity within 2y preceding index pregnancy conception[b]** | | | |
| Mental health-related ED visit or hospitalization | 86 (0.5) | 1248 (16.7) | 1017 (18.7) |
| Non-opioid/multi-drug-related health care | 0 (0.0) | 228 (3.0) | 873 (16.0) |
| Alcohol-related health care | 31 (0.2) | 382 (5.1) | 626 (11.5) |
| Tobacco-related health care | 199 (1.1) | 439 (5.9) | 460 (8.4) |
| HIV or hepatitis at any prior time | 63 (0.3) | 122 (1.6) | 350 (6.4) |
| Pain-related hospital care[c] | 1488 (8.1) | 4795 (64.1) | 1153 (21.2) |
| High medical comorbidity | 1117 (6.1) | 5358 (71.7) | 1345 (24.7) |

© 2022 Camden A et al. *JAMA Network Open.*

| Characteristic, Number (%) | Latent Class<br>Class 1: Short-term analgesia with low comorbidity N=18,313 | Class 2: Pain management with comorbidity N=7478 | Class 3: medication for OUD or unregulated opioid use N=5450 |
|---|---|---|---|
| Asthma within 5y before conception | 182 (1.0) | 316 (4.2) | 99 (1.8) |
| COPD at any prior time | 95 (0.5) | 129 (1.7) | 44 (0.8) |
| Obesity | 572 (3.1) | 781 (10.4) | 50 (0.9) |
| Chronic hypertension at any prior time | 493 (2.7) | 617 (8.3) | 117 (2.1) |
| Diabetes mellitus at any prior time | 551 (3.0) | 765 (10.2) | 169 (3.1) |

a As noted within health care records, which may not be comprehensive.

b Unless otherwise specified.

c Pain conditions include low back pain/abdominal pain, migraine, cancer and chronic pain conditions (rheumatoid arthritis, fibromyalgia, joint pain, chronic pancreatitis, peripheral neuropathy, sickle cell disease, and renal calculi).

OAT opioid agonist therapy; ED emergency department; COPD chronic obstructive pulmonary disease; OUD opioid use disorder; OHIP Ontario Health Insurance Plan

© 2022 Camden A et al. *JAMA Network Open.*

**eTable 4. Characteristics of 31 241 People Who Used an Opioid During Pregnancy Stratified by the 4-Group Solution Identified by the Latent Class Analyses, 2014 to 2019**

| | Latent Class | | | |
|---|---|---|---|---|
| | **Class 1: Short-term analgesia with low comorbidity** | **Class 2: Mixed opioid use & high social and medical needs** | **Class 3: Pain management with comorbidity** | **Class 4: Medication for OUD or unregulated opioid use** |
| **Characteristic, Number (%)** | **N=15,543** | **N=2553** | **N=8945** | **N=4200** |
| **Opioid use in the index pregnancy** | | | | |
| Analgesic prescribed for < 30 days | 14,266 (91.8) | 899 (35.2) | 6788 (75.9) | 12 (0.3) |
| Analgesic prescribed for 30-89 days | 625 (4.0) | 43 (1.7) | 859 (9.6) | 14 (0.3) |
| Analgesic prescribed for 90+ days | 289 (1.9) | 37 (1.4) | 978 (10.9) | 25 (0.6) |
| Methadone OAT only | 25 (0.2) | 505 (19.8) | 125 (1.4) | 1905 (45.4) |
| Buprenorphine OAT only | Suppressed | 144 (5.6) | Suppressed | 655 (15.6) |
| Other OAT | Suppressed | 85 (3.3) | Suppressed | 272 (6.5) |
| OAT + prescribed analgesic | 0 (0.0) | 165 (6.5) | 91 (1.0) | 229 (5.5) |
| Neonatal abstinence syndrome alone | 326 (2.1) | 566 (22.2) | 38 (0.4) | 756 (18.0) |
| Opioid-related hospital care only | 8 (0.1) | 109 (4.3) | 6 (0.1) | 332 (7.9) |
| OAT initiation during pregnancy | 0 (0.0) | 167 (6.5) | 0 (0.0) | 556 (13.2) |
| **Demographics at the index pregnancy conception** | | | | |
| Age in index pregnancy, y | | | | |
| 12-19 | 229 (1.5) | 328 (12.8) | 144 (1.6) | 110 (2.6) |
| 20-24 | 1712 (11.0) | 981 (38.4) | 1157 (12.9) | 880 (21.0) |
| 25-29 | 4002 (25.7) | 660 (25.9) | 2424 (27.1) | 1556 (37.0) |
| 30-34 | 5644 (36.3) | 378 (14.8) | 2862 (32.0) | 1126 (26.8) |
| 35-39 | 3238 (20.8) | 165 (6.5) | 1766 (19.7) | 454 (10.8) |
| 40-50 | 718 (4.6) | 41 (1.6) | 592 (6.6) | 74 (1.8) |
| Neighbourhood Level Income Quintile | | | | |
| Q1 (lowest or unknown) | 3428 (22.1) | 1189 (46.6) | 2671 (29.9) | 2286 (54.4) |
| Q5 (highest) | 2457 (15.8) | 200 (7.8) | 1018 (11.4) | 233 (5.5) |
| Rural residence | 1785 (11.5) | 471 (18.4) | 997 (11.1) | 1286 (30.6) |
| Immigrant or recent OHIP registrant | 2823 (18.2) | 42 (1.6) | 1455 (16.3) | 13 (0.3) |
| 3+ prior livebirths | 1316 (8.5) | 305 (11.9) | 1251 (14.0) | 1101 (26.2) |
| **Social risk factors within 2y preceding index pregnancy conception** | | | | |
| Social risk composite | 218 (1.4) | 998 (39.1) | 295 (3.3) | 793 (18.9) |
| Infant discharged to social services at birth | 56 (0.4) | 380 (14.9) | 64 (0.7) | 455 (10.8) |
| Criminal justice system involvement[a] | 103 (0.7) | 300 (11.8) | 99 (1.1) | 223 (5.3) |
| Homelessness[a] | Suppressed | 205 (8.0) | Suppressed | 39 (0.9) |

© 2022 Camden A et al. *JAMA Network Open.*

| | Latent Class | | | |
|---|---|---|---|---|
| | **Class 1: Short-term analgesia with low comorbidity** | **Class 2: Mixed opioid use & high social and medical needs** | **Class 3: Pain management with comorbidity** | **Class 4: Medication for OUD or unregulated opioid use** |
| Violence-related health care use | 59 (0.4) | 453 (17.7) | 132 (1.5) | 204 (4.9) |
| **Medical morbidity within 2y preceding index pregnancy conception[b]** | | | | |
| Mental health-related ED visit or hospitalization | 111 (0.7) | 1424 (55.8) | 644 (7.2) | 172 (4.1) |
| Non-opioid/multi-drug-related health care | Suppressed | 942 (36.9) | Suppressed | 155 (3.7) |
| Alcohol-related health care | 69 (0.4) | 762 (29.8) | 102 (1.1) | 106 (2.5) |
| Tobacco-related health care | 168 (1.1) | 321 (12.6) | 386 (4.3) | 223 (5.3) |
| HIV or hepatitis at any prior time | 52 (0.3) | 196 (7.7) | 116 (1.3) | 171 (4.1) |
| Pain-related hospital care[c] | 837 (5.4) | 1248 (48.9) | 4969 (55.6) | 382 (9.1) |
| High medical comorbidity | 12 (0.1) | 1673 (65.5) | 5906 (66.0) | 229 (5.5) |
| Asthma within 5y before conception | 159 (1.0) | 111 (4.3) | 303 (3.4) | 24 (0.6) |
| COPD at any prior time | 78 (0.5) | 31 (1.2) | 135 (1.5) | 24 (0.6) |
| Obesity | 437 (2.8) | 50 (2.0) | 889 (9.9) | 27 (0.6) |
| Chronic hypertension at any prior time | 380 (2.4) | 74 (2.9) | 714 (8.0) | 59 (1.4) |
| Diabetes mellitus at any prior time | 404 (2.6) | 86 (3.4) | 882 (9.9) | 113 (2.7) |

a As noted within health care records, which may not be comprehensive.

b Unless otherwise specified.

c Pain conditions include low back pain/abdominal pain, migraine, cancer and chronic pain conditions (rheumatoid arthritis, fibromyalgia, joint pain, chronic pancreatitis, peripheral neuropathy, sickle cell disease, and renal calculi).

Sample sizes <6 were suppressed.

OAT opioid agonist therapy; ED emergency department; COPD chronic obstructive pulmonary disease; OUD opioid use disorder; OHIP Ontario Health Insurance Plan

© 2022 Camden A et al. *JAMA Network Open.*

**eTable 5. Risk of Experiencing Drug Overdose and All-Cause Mortality Within 365 Days After the Index Birth Hospitalization, Shown by the 3, 4 and 5 Group Solutions Identified by the Latent Class Analyses**

Relative risks are adjusted for gestational parent age and year of delivery.

| | | | | Unadjusted | | Adjusted[a] | |
|---|---|---|---|---|---|---|---|
| | Number at risk | Number of events | % with outcome | RR | 95% CI | RR | 95% CI |
| **DRUG OVERDOSE** | | | | | | | |
| **3 Class Model** | | | | | | | |
| Short-term analgesia with low comorbidity | 16,993 | 90 | 0.53 | 1.00 [Ref] | | 1.00 [Ref] | |
| Pain management with comorbidity | 7005 | 145 | 2.07 | 3.94 | 3.00, 5.10 | 3.87 | 2.97, 5.03 |
| Medication for OUD or unregulated opioid use | 4985 | 157 | 3.15 | 6.03 | 4.70, 7.80 | 5.45 | 4.15, 7.15 |
| **4 Class Model** | | | | | | | |
| Short-term analgesia with low comorbidity | 14,425 | 67 | 0.46 | 1.00 [Ref] | | 1.00 [Ref] | |
| Mixed opioid use & high social and medical needs | 2349 | 131 | 5.58 | 12.30 | 9.17, 16.51 | 11.69 | 8.52, 16.03 |
| Pain management & comorbidity | 8381 | 119 | 1.42 | 3.07 | 2.28, 4.14 | 3.17 | 2.35, 4.28 |
| Medication for OUD or unregulated opioid use | 3828 | 75 | 1.96 | 4.25 | 3.06, 5.91 | 4.09 | 2.92, 5.72 |
| **5 Class Model** | | | | | | | |
| Short-term analgesia with low comorbidity | 12,827 | 58 | 0.45 | 1.00 [Ref] | | 1.00 [Ref] | |
| Analgesia in young people | 4299 | 75 | 1.74 | 3.88 | 2.75, 5.46 | 3.42 | 2.33, 5.02 |
| Medication for OUD or unregulated opioid use | 3630 | 70 | 1.93 | 4.30 | 3.04, 6.09 | 4.02 | 2.81, 5.76 |
| Pain management with comorbidity | 6640 | 87 | 1.31 | 2.91 | 2.09, 4.06 | 3.07 | 2.20, 4.29 |
| Mixed opioid use & high social and medical needs | 1587 | 102 | 6.43 | 14.66 | 10.63, 20.24 | 13.64 | 9.71, 19.17 |
| **ALL-CAUSE MORTALITY** | | | | | | | |
| **3 Class Model** | | | | | | | |
| Short-term analgesia with low comorbidity | 16,993 | 11 | 0.06 | 1.00 [Ref] | | 1.00 [Ref] | |
| Pain management & comorbidity | 7005 | 9 | 0.13 | 1.99 | 0.82, 4.79 | 2.02 | 0.82, 4.96 |
| Medication for OUD or unregulated opioid use | 4985 | 26 | 0.52 | 8.07 | 3.99, 16.34 | 7.94 | 3.75, 16.83 |
| **4 Class Model** | | | | | | | |
| Short-term analgesia with low comorbidity | 14,425 | 7 | 0.05 | 1.00 [Ref] | | 1.00 [Ref] | |
| Mixed opioid use & high social and medical needs | 2349 | 14 | 0.60 | 12.31 | 4.97, 30.51 | 13.07 | 4.98, 34.27 |
| Pain management with comorbidity | 8381 | 9 | 0.11 | 2.21 | 0.82, 5.94 | 2.27 | 0.84, 6.11 |
| Medication for OUD or unregulated opioid use | 3828 | 16 | 0.42 | 8.62 | 3.55, 20.96 | 8.77 | 3.58, 21.48 |
| **5 Class Model** | | | | | | | |

© 2022 Camden A et al. *JAMA Network Open.*

| | | | | | | | |
|---|---|---|---|---|---|---|---|
| Short-term analgesia with low comorbidity | 12,827 | Suppressed | | | | | |
| Analgesia in young people | 4299 | Suppressed | | | | | |
| Medication for OUD or unregulated opioid use | 3630 | 16 | 0.44 | | | | |
| Pain management with comorbidity | 6640 | 8 | 0.12 | | | | |
| Mixed opioid use & high social and medical needs | 1587 | 14 | 0.88 | | | | |

Sample sizes <6 were suppressed.

OUD opioid use disorder; RR relative risk; CI confidence interval.

© 2022 Camden A et al. *JAMA Network Open.*

**eFigure 1. Risk of Drug Overdose/Death 365 Days After Birth Hospitalization by Groups Identified by LCA**

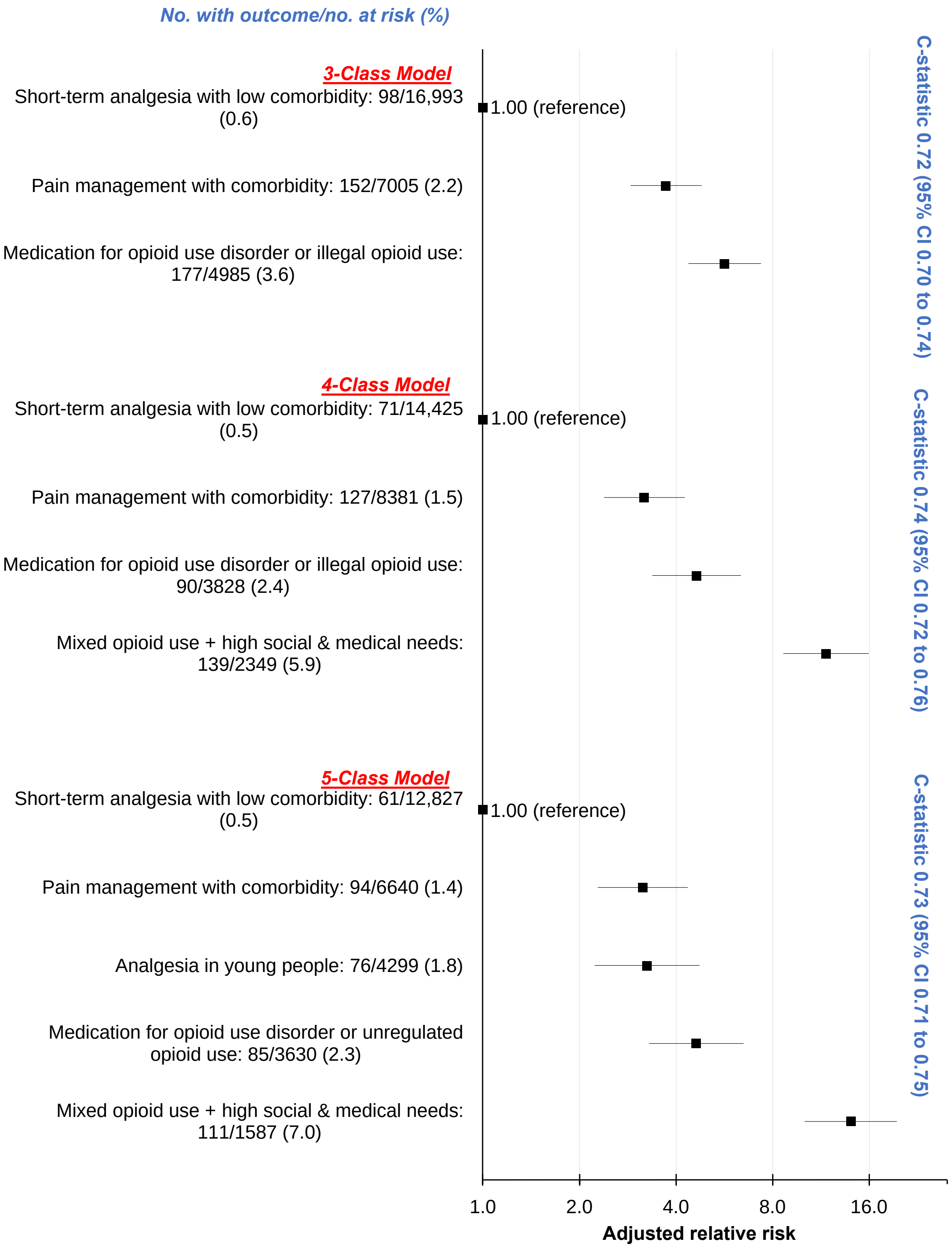


Included are 28,983 people with a birth between January 1, 2014 and June 30, 2019. Relative risks are adjusted for gestational parent agein years and year of delivery. C-statistics were computed using multivariable logistic regression. LCA latent class analysis.

© 2022 Camden A et al. *JAMA Network Open.*
